# Supplementary material for: The ABC transporter Opp imports reduced glutathione, while Gsi imports glutathione disulfide in Escherichia coli
Source: Redox Biol. 2024 Dec 3;79:103453. doi: 10.1016/j.redox.2024.103453 (PMC11719327; doi:10.1016/j.redox.2024.103453)
Supplement: Multimedia component 1 [file mmc1.pdf]

Supplementary Material to:

The ABC transporter Opp imports reduced glutathione, while Gsi imports glutathione disulfide in *Escherichia coli*

Lisa R. Knoke, Maik Muskietorz, Lena Kühn and Lars I. Leichert

Ruhr University Bochum, Institute for Biochemistry and Pathobiochemistry – Microbial Biochemistry, Bochum, Germany.

*Correspondence to Lars I. Leichert: lars.leichert@ruhr-uni-bochum.de

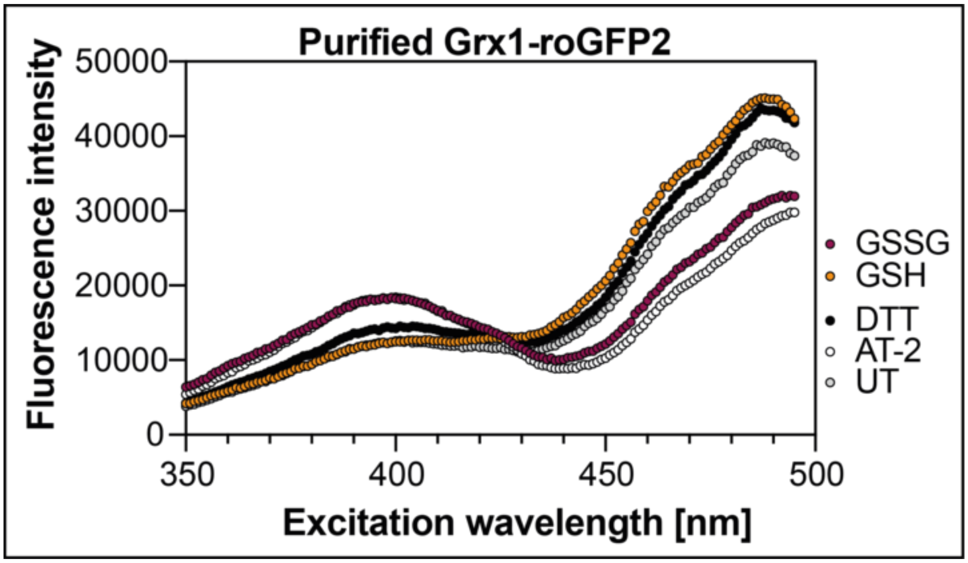

Figure S1: Test of commercial glutathione preparations used in this study for contamination with GSSG (in the case of GSH) or GSH (in the case of GSSG. Neither the GSH nor the GSSG preparation elicits meaningful probe oxidation or reduction when compared to DTT or AT-2, respectively.

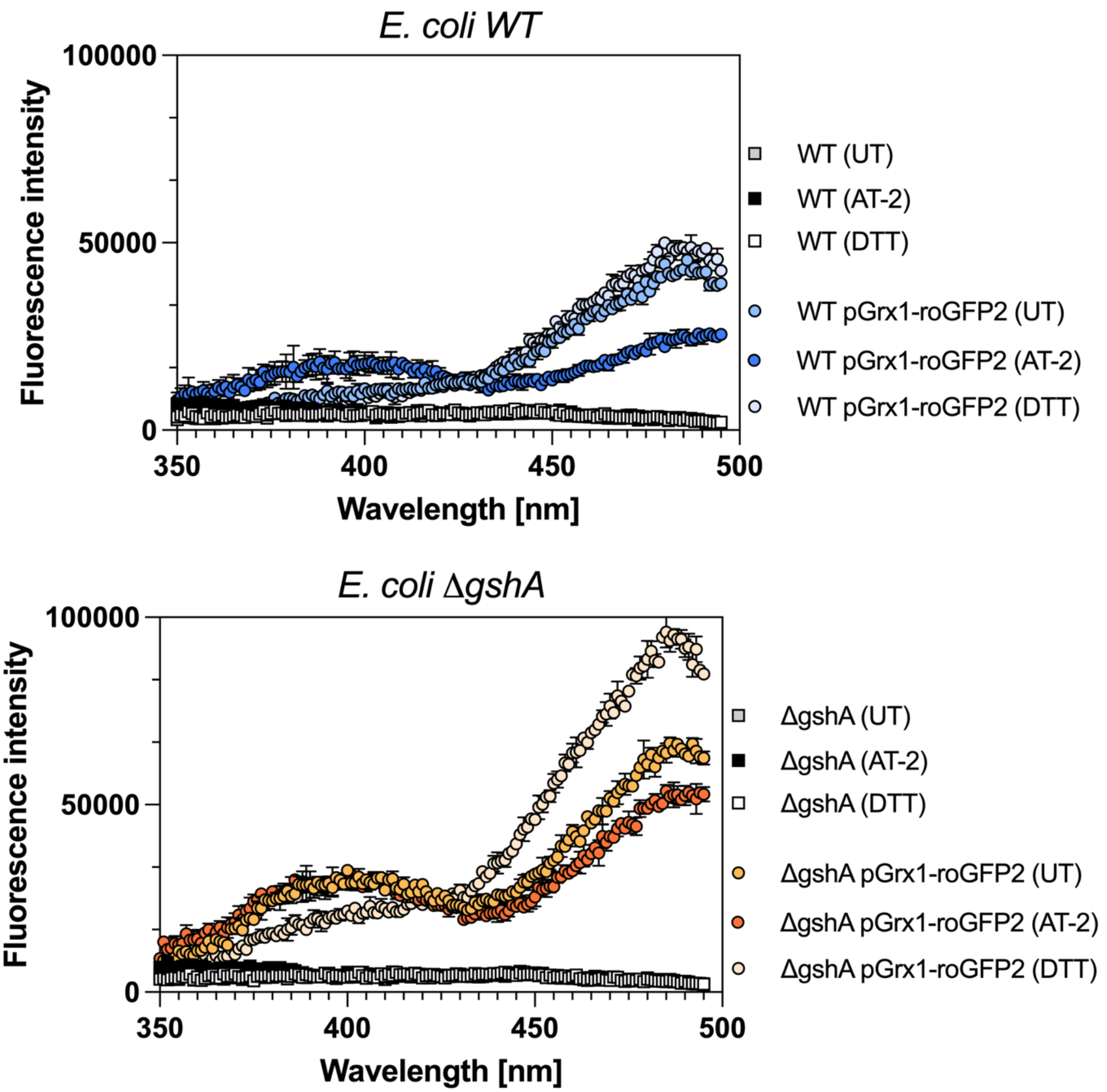

Figure S2: Autofluorescence intensity in *E. coli* in comparison to intracellular probe fluorescence intensity. Excitation spectra over the range of 350-500 nm at 510 nm emission were recorded in different *E. coli* strains with and without a Grx1-roGFP2-expression plasmid in the instrument used in our study under the conditions used in our study. The observed autofluorescence in the respective parental strains was significantly lower than the probe signal from strains carrying a a Grx1-roGFP2-expression plasmid.
